# 

**Published:** 1822-06

**Authors:** 


### MONTHLY CATALOGUE OF MEDICAL BOOKS.

A Case of Transverse Fracture of the Patella. By George Fielding. 1s.

The Quarterly Journal of Foreign Medicine and Surgery, and of the Sciences connected with them. No. XIV. 8vo. 4s. 6d.

The Nursery Guide; or, Remarks on the Conduct of a Nursery: containing Information likely to be useful to those who may shortly take upon themselves the Duties of a Mother; and particularly offered to the Notice of Females in general. By Henry Thompson, Surgeon, Enfield, Middlesex. 8vo.

An Inquiry into the Comparative Forces of the Extensor and Flexor Muscles connected with the Joints of the Human Body. By Julius Jeffreys, M.R.C.S. 8vo. 2s. 6d.

Chapman's Elements of Therapeutics and Materia Medica. Vol. I. 8vo. 16s.

Tracts on Medical Jurisprudence; with a Preface, Notes, and a Digest of the Law relating to Insanity and Nuisance. By Thomas Cooper, M.D. 8vo. 18s.

A Memoir on Contagion, more especially as it respects the Yellow Fever. By N. Potter, M.D. 8vo. 6s.

A Practical and Historical Treatise on Consumptive Diseases, deduced from original Observations, and collected from Authors of all Ages. By Thos. Young, M.D. F.R. and L.S. &c. 8vo. 12s. boards.

The Pathology of Fever; being the Subject of the Gulstonian Lecture lately delivered at the Royal College of Physicians. By J. R. Park, M.D. Member of the Royal College of Physicians. 8vo. 6s. boards.

### TO CORRESPONDENTS.

*We have received several valuable* Communications, *and* new Books *for review, which shall meet with our earliest attention.*

